# The Significance of Selecting an Appropriate Patient-Reported Outcome Measure (PROM): A Cross-Cultural Adaptation of the Specific Paediatric International Documentation Committee Subjective (Pedi-IKDC) Knee Form

**DOI:** 10.3390/children10121930

**Published:** 2023-12-14

**Authors:** Viktorija Brogaitė Martinkėnienė, Donatas Austys, Andrius Šaikus, Andrius Brazaitis, Giedrius Bernotavičius, Aleksas Makulavičius, Gilvydas Verkauskas

**Affiliations:** 1 Faculty of Medicine, Vilnius University, LT-03101 Vilnius, Lithuania; 2 Department of Children’s Orthopedics and Traumatology, Vilnius University Hospital Santaros Klinikos, LT-08406 Vilnius, Lithuaniagiedrius.bernotavicius@santa.lt (G.B.); 3 Department of Public Health, Institute of Health Sciences, Faculty of Medicine, Vilnius University, LT-03101 Vilnius, Lithuania; donatas.austys@mf.vu.lt; 4 Centre for Radiology and Nuclear Medicine, Vilnius University Hospital Santaros Klinikos, LT-08661 Vilnius, Lithuania; 5 Clinic of Gastroenterology, Nefrourology and Surgery, Faculty of Medicine, Vilnius University, LT-03101 Vilnius, Lithuania; 6 Clinic of Rheumatology, Orthopaedics Traumatology and Reconstructive Surgery, Faculty of Medicine, Vilnius University, LT-03101 Vilnius, Lithuania

**Keywords:** patient-reported outcomes measures, Pedi-IKDC, cross-cultural adaptation, children, knee injuries, psychometric properties

## Abstract

Introduction: The selection of an appropriate PROM is a crucial aspect in assessing outcomes. Questionnaires that have not been designed or validated for a paediatric population are routinely used. Using a questionnaire requires translation, cultural adaptation, and testing the psychometric properties of the translated questionnaire. There is no applicable questionnaire in our country for children with knee-specific conditions in sports orthopaedics. Therefore, this study aims to translate, culturally adapt, and assess the psychometric properties of the Paediatric IKDC (Pedi-IKDC) questionnaire within the Lithuanian paediatric population. Methods: The translation was conducted in accordance with international standards. Patients aged 11–17 years with various knee disorders participated in three surveys and completed the Pedi-IKDC, Lysholm, and PedsQL questionnaires. Interviews with patients following the translation process, in addition to floor and ceiling effects, were used to assess content validity. Cronbach alpha (α) statistics and the intraclass correlation coefficient (ICC) were applied to measure internal consistency and reproducibility, respectively. The standard error of measurement (SEM) and smallest detectable change (SDC) were calculated to assess reliability. Pearson correlations were calculated between Pedi-IKDC and Lysholm PedsQL scores to determine criteria validity. The effect size (ES) and standardised response mean (SRM) were calculated to assess the responsiveness to change. Results: Cronbach’s alpha (α) was 0.91 for the total score, 0.75 for symptoms, and 0.92 for the sport/function component. The ICC for overall scores was 0.98, with each question ranging from 0.87 to 0.98. The SEM was 2.97, and the SDC was 8.23. Lysholm and PedsQL physical functioning domain scores had moderate correlations (0.8 > r > 0.5), and the overall PedsQL score had a weak correlation (0.5 > r > 0.2) to the Pedi-IKDC score. The floor and ceiling effects were 3.3% and 1.6%, respectively. The SRM was 1.72 and the ES was 1.98. Conclusions: The Lithuanian Pedi-IKDC version is an appropriate evaluation instrument for assessing outcomes in children with knee disorders. All of the psychometric features produced acceptable results.

## 1. Introduction

Patient-reported outcomes measures (PROMs) have grown in significance for clinical research and care evaluation [1,2]. PROMs are self-report measures that advocate the collection of data on constructs provided by patients without interpretation by third parties [1]. These instruments come in a variety of types for various populations and aims [3]. In orthopaedics, PROMs are most frequently used to assess a patient’s physical and mental condition prior to or after an intervention [1,4]. As with massive consumption, the PROMs subject has raised several issues [2]. The first current concern with PROMs is the appropriate selection of the health assessment instrument for the research [1]. Primarily, the PROM chosen should be appropriate for the population and the state of the research in order for the clinician or researcher interpreting clinical findings to avoid the risk of making an incorrect suggestion or carrying out an unneeded intervention [2]. Second, PROMs must have trustworthy measuring properties [1,2,4]. The most widely used criteria for testing the measurement properties of PROMs are established by experts in the Consensus-Based Standards for the Selection of Health Status Measurement Instruments (COSMIN) publication [1,5,6].

The knee joint is the most commonly injured joint, particularly in the area of sports orthopaedics [7,8]. There are mainly two categories of knee-specific PROMs, which are differentiated by knee conditions and the target population. The Knee Injury and Osteoarthritis Outcome Score (KOOS), Western Ontario and McMaster Universities Osteoarthritis Index (WOMAC), Oxford Knee Score (OKS), and New Knee Society Score (KSS) are scores that are widely used and designed for knee osteoarthritis, and total knee arthroplasty outcomes are used for measurement [9,10]. Because they were designed for osteoarthritis patients, these instruments are not approved to be used in patients experiencing sports knee injuries such as ligament or meniscal tears [2]. The other category of knee-specific PROMs has been developed for sports-related knee injuries [11]. Therefore, the International Knee Documentation Committee Subjective Knee Form (IKDC) is one of the most often used patient-reported outcome measures in orthopaedic sports injury research and is specifically developed for ligament, meniscal, and cartilage injuries [12]. The IKDC score has been validated several times for evidence of its relevance, even among adolescents [12,13,14,15,16]. So far, numerous countries have made translations and cultural adaptations of the IKDC score, with the overall goal of having many countries use the same scale for the same conclusions and communication [17,18,19]. Eventually, knee injuries and knee-related surgery have thus become more common in children, who now participate in more professional and intense sports and physical activities [20,21,22]. As a result of current issues with the appropriateness of PROMs, some authors have determined that the adult-developed IKDC questionnaire is not suited for children due to their incomprehension of the items [22,23,24,25]. This led to the development of a modified paediatric version of IKDC questionnaire [23,26]. Even with normative data research, the Pedi-IKDC score is developed and validated in the USA as a reliable tool for children with knee disorders and has been translated into several languages [27,28,29,30,31]. However, questionnaires that have not been designed or validated for a paediatric population are frequently utilised in studies [32]. In our country, there is no applicable questionnaire for children with knee-specific conditions following knee injury. According to current concerns about PROMs, using a questionnaire requires translation, cultural adaptation following international criteria, and testing the psychometric properties of the translated questionnaire [4]. Therefore, this study aims to translate, culturally adapt, and assess the psychometric properties of the Pedi-IKDC questionnaire within the Lithuanian paediatric population.

## 2. Methods

Ethics: This study was approved by the hospital’s Ethics Committee and Vilnius Regional Biomedical Research Ethics Committee, Number 2021/5-1353-825. All participant’s parents or caregivers gave informed consent prior to inclusion in the study. Patient care was not affected by participation in this study.

### 2.1. Translation Procedure

The translation and cultural adaptation process was performed in accordance with international guidelines [33,34]. Upon receipt of the copyright agreement, two independent translators, native Lithuanian speakers, one of whom was a professional English translator, completed a forward translation. Subsequently, the author of this study and both translators combined two distinct translation versions to create the first Lithuanian version. To ensure the accuracy of the forward translation and to properly evaluate the meanings in both languages, the backward translation was conducted by two distinct native English speakers who had a strong command of the Lithuanian language. The Expert Committee, which included the main study author, two orthopaedic surgeons, translators who provided forward and backward translations, and one independent medical practitioner from an outpatient clinic, compared these two translations to the original version. The conclusions of the Expert Committee were used to create a further Lithuanian version of the questionnaire. The pre-final version was tested by 10 children (6 boys and 4 girls) with different knee conditions who were asked to comment on all the questions and answers when completing the score and to assess any incomprehension. The piloting testing procedure led in word modifications in items 7 and 8 prior to the launch of the final version to improve understanding of the distinctions between these items.

### 2.2. Clinical Study

Patients were recruited at Vilnius University Santaros Clinics Children’s Hospital from March to August 2021. The inclusion criteria were paediatric patients with knee disorders and patients whose parents had given their consent after having familiarised themselves with all the information provided about this study. Intellectually disabled people and non-Lithuanian speakers were excluded. The protocol for the study’s conduct has received ethical approval from the Vilnius Regional Biomedical Research Ethics Committee (Number 2021/5-1353-825). Selected patients of the outpatient clinic or who were hospitalised in the children’s orthopaedic and traumatology department were asked to complete the Lithuanian Pedi-IKDC version along with the Lysholm Knee score and PedsQL generic (health-related quality of life) score (survey A). Patients were encouraged to submit the questionnaires on their own, but where necessary, parents or legal guardians were allowed to assist their children.

All participants were contacted via phone two weeks later (a mean of 14 days ± 2.9 range 9–21 days) and asked if their knee symptoms or functionality changed from the previous survey. Following the negative response, only patients with stable knee issues were requested to resubmit the identical Pedi-IKDC form via email (survey B). The time interval of 9–21 days was selected to minimise patients’ memory of their prior response and to avoid any change in the condition of their knee. The final survey (survey C) was provided to participants who had knee-related treatment (conservative or surgical). These patients completed the Pedi-IKDC during a live consultation within 4 months (mean, 17.48 ± 3.6 weeks; range 9–25 weeks) after treatment. The time point average of 4 months was selected due to the expectation of potential clinical changes in the patients This study was intended to involve more distinct knee-condition cases, as this is beneficial for a questionnaire cultural adaption process.

### 2.3. Questionnaires

Pedi-IKDC is used to measure knee-related symptoms, function, and sports activity among children. The questionnaire consists of 13 items, each of which is scored using one of three rating systems: a range of 0 to 10 for items 2, 3, 12, and 13, a range of 0 to 4 for items 1, 4, 5, 6, 9, and 10, and a range of 0 to 1 for items 7 and 8. The 11th item consists of nine subquestions, each of which can be scored from 0 to 4. The overall score value is calculated by adding only the responses to 12 items and dividing the total by 92 (the highest number of points attainable). The score runs from 0 (worst case scenario) to 100 (best case scenario). The Pedi-IKDC has been proven to be a valid, trustworthy, and responsive questionnaire in a paediatric population with varied knee disorders, especially ligament and meniscal injuries, joint instability, and other disorders [20,22,24,25,26].

The Lysholm knee scoring scale was originally developed to assess knee symptoms of instability for patients with knee ligament pathology [35]. Over time, it has proven to be suitable for a variety of knee pathologies and also for the adolescent population [36,37,38]. The scale comprises eight items. Higher values of the score indicate better functioning of the knee (range from 0 to 100).

The PedsQL (health-related paediatric quality of life) generic score estimates the quality of life of paediatric patients. The questionnaire consists of four domains with 23 questions: general physical functioning, emotional, social, and functioning at school domains [39].

### 2.4. Psychometric Properties

Psychometric properties were assessed using the classical test of theory (CTT) in accordance with the standards of the health measurement instrument [5,6,40]. Test–retest reliability represents whether it provides similar results when repeated under stable conditions [6,40]. It was determined between the scores of survey A and survey B and was obtained by calculating the interclass correlating coefficient (ICC) for the overall Pedi-IKDC scale and for the separate items using the two-way random model in absolute agreement. An ICC above 0.70 is considered acceptable. The Standard Error of measurement (SEM) was detected using an equation (*SEM* = *SD* ∗ √1 − ICC) indicating the measurement error in the group. The smallest detectable change (SDC), determined via equation (1.96 ∗ √2 ∗ *SEM*), shows a statistically significant change between the two measurements and indicates a measurement error at the individual level [40]. Internal consistency demonstrates the homogeneity between the items of the questionnaire [40]. It was assessed by calculating the Cronbach alpha coefficient for responses to survey A. An a value > 0.7 was considered acceptable. Criterion validity was estimated by assessing the correlation between the Pedi-IKDC and Pedi-IKDC components of scores and the Lysholm, PedsQL scale, PedsQL subscales [40]. In addition, the separate items of Pedi-IKDC were compared to those from Lysholm, the PedsQL overall score, and domains of PedsQL. The Pearson correlation coefficient (r) was used to determine the relationship.

First, content validity was tested by performing an interview in the patient-based pilot research for the incomprehensibility of individual items and by assessing the floor and ceiling effects of survey A score values [40,41]. The floor effect is the percentage of patients who obtained the lowest possible score (from 0 to 10) and the ceiling effect is the percentage of patients who obtained the highest possible score (from 90 to 100). They were considered acceptable provided they were less than 30%. Responsiveness is the power to change over time or after treatment. It was evaluated by comparing Pedi-IKDC scores of surveys A and C. The effect size (ES) was calculated using the following equation: mean survey C- mean survey A/standard deviation survey A, and standardised response mean (SRM): mean survey C- mean survey A/standard deviation difference (survey C- survey A). The effect size and standardised response means of 0.2, 0.5, and 0.8 were considered small, medium, or large, respectively [40].

### 2.5. Statistics

Data were analysed with SPSS version 25 (IBM SPSS) and Microsoft Excel programs. A level of significance < 0.05 was applied for statistical analysis. Using SPSS Soft, the values of internal consistency, test–retest reliability, and criterion validity were determined. The calculation of the SEM, SDC, EF, and SRM is performed utilising the equations stated above. In Excel, the floor and ceiling effects are generated. In accordance with the literature, a sample size of a minimum of 50 patients is acceptable for adaptation and validation reliable process [42].

## 3. Results

The translation and cultural adaptation process was performed under described international rules. Firstly, the discussion was on the word “activities” in items 1, 6, 9, and 10. This word was replaced with “action” in the backward translation therefore the conclusion was made as to which word was more suitable. Secondly, the modification in the expression “hurts so much, I can’t stand it” on item 3 was made as closely to the Lithuanian language as possible. In item 4, the phrase “bending and moving” was replaced with “bending” only as the previously used words have the same meaning in the Lithuanian language when referring to the knee functioning. The most complicated expression and translation process was in item 9, “the feeling like the knee can’t hold you up”. The translation was made into the expression that “it could not hold you on the legs and not functioning”. “Most of the time” in item 10 was changed into the “regularly doing something” expression. “Soccer” and “heavy lifting” in items 1, 6, 9, and 10 were replaced with “football” and “weight training”, respectively. The content validity of each item was evaluated by asking patients if there was any incomprehensibility. Due to a misunderstanding by questioned patients, the word “moving” was changed to “bending” in items 7 and 8. The final Lithuanian version of the Pedi-IKDC is attached in Supplementary Materials.

### 3.1. Demographic

Pedi-IKDC, Lysholm, and PedsQL forms were completed by 60 patients during Survey A, 57 patients during Survey B, and 42 patients during Survey C. Table 1 shows the demographic information of the patients in this study.

### 3.2. Internal Consistency/Reliability

Cronbach’s alpha was larger than 0.7 in both the total score and in individual components of the score (Table 2). Table 3 shows the test–retest reliability results for the overall Pedi-IKDC score. The interclass correlation coefficient for the score’s individual items was more than 0.70. Items 7, 8, and 10 had the lowest ICC values (Table 4). SEM and SDC values were 2.32 and 8.23, respectively.

The additional assessment of content validity was the floor and ceiling effects, which were 3.3% and 1.6%, respectively.

### 3.3. Criterion Validity

**Figure 1:**
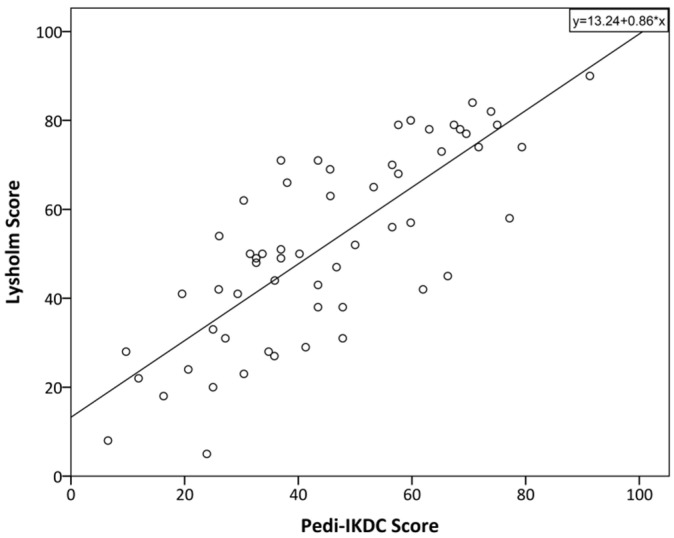
The relationship between Lysholm and Pedi-IKDC scores of the participants of this study. The line represents linear regression; R^2^ = 0.622.

**Figure 2:**
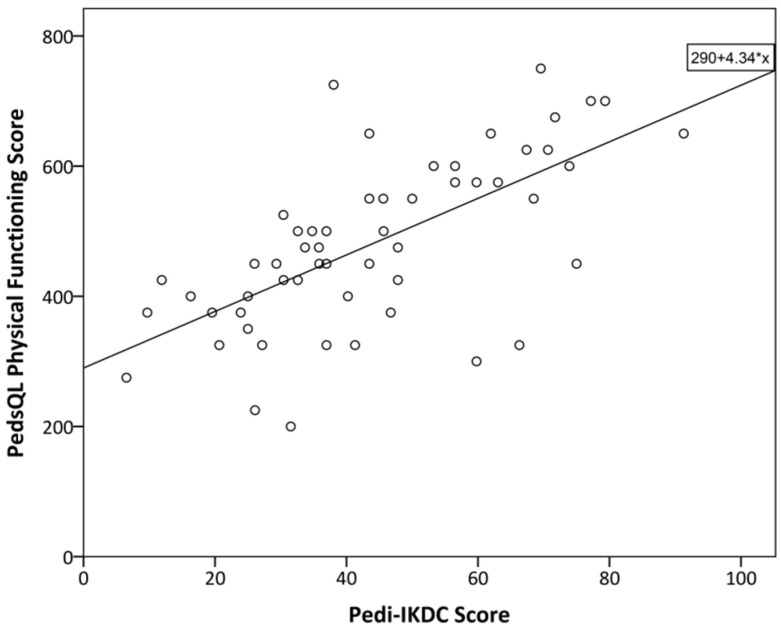
The relationship between PedsQL physical functioning and Pedi-IKDC scores of the participants of this study. The line represents linear regression; R^2^ = 0.428.

**Figure 3:**
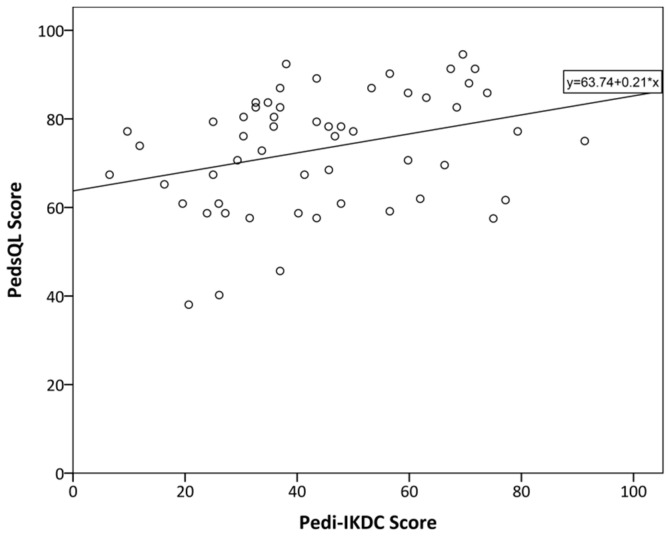
The relationship between general PedsQL and Pedi-IKDC scores of the participants of this study. The line represents linear regression; R^2^ = 0.135.

Significantly (*p* < 0.05) moderate (0.8 > r > 0.5) correlations were found between the overall Pedi-IKDC score, its components (symptoms, sport, and function), the Lysholm score, and the PedsQL physical functioning domain (see Figure 1 and Figure 2). Further significantly weak (0.5 > r > 0.2) correlations were found between Pedi-IKDC and overall PedsQL scores (see Figure 3) (Table 5). As regards other PedsQL domains (social, emotional, and school functioning), there were no significant correlations with the Pedi-IKDC score. In addition, the relations between separate items of the Pedi-IKDC and overall scores of the Lysholm and PedsQL physical functioning domain revealed significantly weak to moderate correlations (0.3 < r < 0.7). Moreover, there were only a few significantly weak correlations (0.2 < r < 0.4, *p* < 0.05) between 4, 6, 11 c, 11 d, and 11 i items and the overall PedsQL score.

Responsiveness was determined by comparing the mean Pedi-IKDC scores of Survey A and Survey C respondents who completed the last form 4 months (mean, 17.48 ± 3.6 weeks; range 9–25 weeks) after receiving treatment, depending on the knee disorder. Briefly, 42 patients completed the Pedi-IKDC form during Survey C. Pedi-IKDC demonstrated a large (>0.80) effect size (82.98–43.72/19.82 = 1.98) and a large (>0.80) standardised response mean (82.98–43.72/22.8 = 1.72).

## 4. Discussion

A cultural and linguistic adaptation of the Pedi-IKDC questionnaire for Lithuanian paediatric patients with knee disorders was created and its psychometric properties were evaluated. So far, there are few validated PROMs in orthopaedic and even knee-addressed conditions in children [4,20,25,26,43,44,45]. The fundamental issue is that many studies do not use appropriate PROMs for children or use them without validation [32,46]. This issue has been explicitly addressed by Phillips et al., Arguelles, and their co-authors in their review studies of patient-reported outcome measures utilised in a paediatric population. According to Dietvorst et al., children’s incorrect comprehension of questions intended for adults may lead to irrelevant outcomes and conclusions [24]. To date, Pedi-IKDC is the most studied and should be preferred over other PROMs [24,25,31]. In their scoping review on paediatric populations, Zebis et al. identified three primary questionnaires that are commonly used to assess outcomes following an anterior cruciate ligament (ACL) injury and concluded that Pedi-IKDC is the only tool that encompasses all three ICF categories (the dimensions of the International Classification of Functioning, Disability, and Health) and is widely utilised to assess outcomes after different knee operations, such as ACL reconstructions [25]. Furthermore, Dietvorst et al. conducted a systematic review and determined that Pedi-IKDC is superior to KOOS-Child as a measurement tool. This conclusion is based on the fact that Pedi-IKDC has been tested in a greater number of research studies and evaluations for its psychometric properties, in comparison to KOOS-Child [24]. In addition, van der Velden et al. performed a study on the Pedi-IKDC and KOOS-Child scores and determined that Pedi-IKDC had superior psychometric properties, making it a better instrument for evaluating knee function in children [31]. However, separate items of Pedi-IKDC have been criticised [43]. Another significant issue presently is the validation technique, which is not sufficiently standardised and is currently being debated [2,6,33,47]. The COSMIN checklist (Consensus-Based Standards for the selection of health status Measurement Instruments) was developed in an international Delphi study to evaluate the methodological quality of studies on measurement properties of health-related patient-reported outcomes (HR-PROs) [5,6,40]. Also, the same group of experts recently made update recommendations on content validity evaluation and provided a revised checklist for systematic reviews of studies with PROMs evaluation [41,48]. This methodology is the classical way and has been used in almost all studies when developing or translating needed PROMs, as was the case in our study. Similarly, a new modern theory of assessing the psychometric properties of PROMs was recently given, which focuses on construct validity in different ways of statistical analyses [47].

For the time being, the Pedi-IKDC is available in English [26] original, Danish [30], Dutch [31], Italian [28], and Spanish [29].

Due to the COSMIN checklist and other authors, content validity is very important, and it determines whether or not an instrument accurately represents the characteristic being assessed based on expert consensus judgment or the measurement of floor and ceiling effects [5,6,40,41]. As to the updates on content validity, the only expert’s consensus should be added with a cognitive interview study or other pilot test performed to evaluate the comprehensiveness and comprehensibility of the PROM [41]. The patient’s view on the comprehension of questions is of greatest significance in light of the recent recommendations [41]. This recommendation is given more for developing PROMs and not for existing ones, as it was followed by Iversen et al. when Pedi-IKDC was developed [23]. In addition, ten patients were also interviewed after the backward translation procedure in our study and the conclusions on comprehensibility were made. Furthermore, the floor and ceiling effects obtained in our study were higher but still acceptable. This might be have been conditioned by the wider marginal values chosen in our study. The Lithuanian version of Pedi-IKDC demonstrated a large effect size (>0.8) and a large standardised response mean (>0.8). These values are close to those of the studies of Kocher et al. (original) and van der Velden et al. [31]. The large effect size and high standardised response indicate that the instrument is responsive [6,40].

The Lithuanian version of Pedi-IKDC demonstrated acceptable internal consistency. The Cronbach alpha coefficient obtained in our study was found to be the same as that in the original study by Kocher et al. [26] and very similar to that in other transcultural adaptation studies [28,30,31]. To obtain a better indicator of internal consistency, the separate components of the score were evaluated. The internal consistency of the component of symptoms (items 1 to 9) and the component of sport/function (items 10 to 13, 11 subitems) were checked by calculating the Cronbach α coefficient. Both components of the score demonstrated acceptable results. The presence of a lower value (α = 0.75) of the first component of the score could be due to the lower number of items compared to the number of questions in the sport/function component. It is known that the Cronbach α coefficient is sensitive to the number of items. The outcome measure tool is reliable when it produces similar results in patients with stable conditions. Considering the overall score’s test–retest reliability, the ICC value obtained in our study was excellent and very close to that of the study by Macchiarola et al. [28]. In addition, the test–retest reliability for separate items was verified. All values obtained were acceptable and higher than 0.7, which indicates good Lithuanian Pedi-IKDC version reproducibility among children with stable knee conditions. The SEM and SDC values in the study were close to those presented in other studies [26,28,30,31]. The higher the values, the greater the discrepancies, which are required for establishing that a true change has occurred [25]. As previously stated by Kocher et al., criterion validity assesses an instrument’s relationship to an accepted outcome instrument—ideally, a gold standard, if one exists [8]. To our knowledge, there is no common agreement on a general health instrument to be used as the “gold standard”. Pedi-IKDC was compared to the widely used Lysholm knee score and the Paediatric quality of life score to assess criterion validity.

Originally, the Lysholm knee score was developed for instability of the knee evaluations in adults, and it is not the ideal choice for children, but correlations were obtained that were still enough to be significantly moderate and strong in our study. In accordance with our expectations, the new Pedi-IKDC was significantly moderately correlated with the physical functioning domain of PedsQL but not with the other domains of this score. This is because Pedi-IKDC is designed to evaluate knee-specific symptoms, function, and sports activity, not the impact of knee disorders on emotional and social health. Different authors have chosen different scores to compare to Pedi-IKDC score, but they all represented mostly moderate to weak correlations between the scores [26,28,29,30,31].

The primary highlight of this study is that translation and linguistic adaptation were carried out in accordance with the internationally norms established by Beaton et al. [34]. In accordance with the literature review and counsel, all of the recommendations were followed during the translation process in our study [33]. Moreover, most surveys of this research were conducted during live consultations so we could control how the patients completed the questionnaires on their own as recommended. Only the second survey was conducted via email, which is also an acceptable method to perform the surveys [49].

Our study’s sample size (N = 60) was smaller than that used in previous research [26,28,29,30,31]. Nevertheless, according to the literature, it was sufficient [42,50]. Based on our findings (the width of the 95% confidence interval of the ICC_agreement_ was 0.22, the correlation between repeated scores was r = 0.8, the expected variances of score = 100, and there was no systematic difference between raters) and the literature, a powerful sample size of 50 cases might be used [42].

Moreover, there was a lack of population heterogeneity in our study; there were older patients (median age was 15 years), similarly to a Dutch validation study, and more ACL and meniscus rupture knee conditions (71.3%) compared to other disorders of the knee [31]. These factors could impact the score values and influence the results of psychometric parameters. On the other hand, the older a population is, the greater a probability exists of patients completing the questionnaire by themselves, according to the recommendations of the questionnaire.

## 5. Conclusions

The psychometric properties of the Lithuanian Pedi-IKDC were deemed sufficient. These findings indicate that the adaption process was conducted competently. This study produced an instrument that is substantially identical to the original and is suitable for clinical research and everyday clinician work in the paediatric population with diverse knee disorders to monitor outcomes.

## Figures and Tables

**Table 1:** Demographic data of the patients.

**Sex**	
F	31 (51.7%)
M	29 (43.8%)
**Age**	
median	15 (range 11–17 years)
SD	1.7
**Diagnosis**	**N (%)**
ACL rupture	5 (8.3%)
Meniscus tear	26 (43.3%)
ACL and meniscus tear	12 (20%)
Patella instability	2 (3.3%)
Osteochondritis dissecans	2 (3.3%)
Osgood Schlatter disease	4 (6.7%)
PCL rupture	1 (1.7%)
Bone cyst	1 (1.7%)
Exostosis	1 (1.7%)
Patellofemoral pain syndrome	3 (5%)
Tibial eminence fracture	1 (1.7%)
MCL rupture	1 (1.7%)
Meniscus root tear	1 (1.7%)

Abbreviations: N—number of patients; SD—standard deviation; F—female; M—male; ACL—anterior cruciate ligament; PCL—posterior cruciate ligament; MCL—medial collateral ligament.

**Table 2:** Internal consistency of Survey A (N = 60).

Score/Component of Pedi-IKDC	α
Pedi-IKDC	0.91
Pedi-IKDC Symptoms	0.75
Pedi-IKDC Sport and Function	0.92

Abbreviations: N—number of patients; Pedi-IKDC—Paediatric International Knee Documentation Committee Subjective Knee Evaluation Form; α—Cronbach’s α coefficient.

**Table 3:** Test–retest reliability in Surveys A and B (N = 57).

Mean Score A (SD)	Mean Score B (SD)	Mean Difference (SD)	ICC	95%CI	SEM	SDC
45.56 (19.34)	42.95 (19.18)	1.9 (4.2)	0.98	0.97 to 0.99	2.32	8.23

Abbreviations: N—number of patients; SD—standard deviation; ICC—interclass correlation coefficient; CI—confidence interval; SEM—standard error of measurement; SDC—smallest detectable change.

**Table 4:** Test–retest interclass correlation coefficient for the separate questions in Surveys A and B (N = 57).

Questions	ICC (95%CI)
1 question	0.94 (0.9 to 0.96)
2 question	0.98 (0.97 to 0.99)
3 question	0.97 (0.96 to 0.98)
4 question	0.91 (0.85 to 0.95)
5 question	0.89 (0.82 to 0.94)
6 question	0.97 (0.95 to 0.98)
7 question	0.88 (0.8 to 0.93)
8 question	0.88 (0.79 to 0.93)
9 question	0.95 (0.91 to 0.97)
10 question	0.87 (0.76 to 0.92)
11 question	0.98 (0.95 to 0.98)
13 question	0.97 (0.95 to 0.98)
11 a subquestion	0.94 (0.89 to 0.96)
11 b subquestion	0.92 (0.86 to 0.96)
11 c subquestion	0.95 (0.91 to 0.97)
11 d subquestion	0.98 (0.97 to 0.99)
11 e subquestion	0.94 (0.88 to 0.96)
11 f subquestion	0.95 (0.91 to 0.97)
11 g subquestion	0.97 (0.95 to 0.98)
11 h subquestion	0.97 (0.95 to 0.98)
11 i subquestion	0.98 (0.97 to 0.99)

Abbreviations: N—number of patients; ICC—interclass correlation coefficient; CI—confidence interval.

**Table 5:** Criterion validity of Pedi-IKDC in Survey A. Correlations to the Lysholm knee score, PedsQL score, and PedsQL physical functioning domain (N = 60).

	Score	Pearson (*r*)	*p* Value
Pedi-IKDC	Lysholm	0.78	0.00
	PedsQl	0.27	0.03
	PedsQL physical functioning	0.65	0.00
Pedi-IKDC symptoms	Lysholm	0.72	0.00
	PedsQL	0.26	0.05
	PedsQL physical functioning	0.57	0.00
Pedi-IKDC sports/function	Lysholm	0.73	0.00
	PedsQL	0.26	0.04
	PedsQL physical functioning	0.67	0.00

Abbreviations: N—number of patients; Pedi-IKDC—Paediatric International Knee Documentation Committee Subjective Knee Evaluation Form; PedsQL—health related paediatric quality of life generic score; Lysholm—Lysholm knee scoring form; Pearson *r*—correlation coefficient.

## Data Availability

The data presented in this study are available on request from the corresponding author: viktorija.brogaite@santa.lt. The data are not publicly available due to study protocol regulations.

## Supplementary Materials

The following supporting information can be downloaded at https://www.mdpi.com/article/10.3390/children10121930/s1. Supplementary file contains the translated Lithuanian Pedi-IKDC form.
