# Supplementary material for: The Significance of Selecting an Appropriate Patient-Reported Outcome Measure (PROM): A Cross-Cultural Adaptation of the Specific Paediatric International Documentation Committee Subjective (Pedi-IKDC) Knee Form
Source: Children (Basel). 2023 Dec 14;10(12):1930. doi: 10.3390/children10121930 (PMC10742502; doi:10.3390/children10121930)
Supplement: Supplementary file 1 [file children-10-01930-s001.zip › children-2754765-supplementary.pdf]

Pedi-IKDC subjektyvus kelio sąnario būklės įvertinimo klausimynas – PACIENTO KLAUSIMYNAS

BENDROJI INFORMACIJA

1. Tyrimo ID: __ __ __

2. Užpildymo diena: __ __ / __ __ / __ __ __ __
MMMM MM DD

APKLAUSA

Kelio traumos data: __ __ / __ __ / __ __ __ __
MMMM MM DD

Norėtume sužinoti daugiau apie tavo traumuotą kelio sąnarį. Kiekvienas klausimas prašo pateikti skirtingą informaciją apie tavo traumuotą kelio sąnarį. Prašome atsakyti į visus toliau pateiktus klausimus.

SIMPTOMAI

1. Jei tavęs paprašytų atlikti toliau nurodytus veiksmus, nurodyk intensyviausius veiksmus, kuriuos galėtum atlikti šiandien, nesukeliant stipraus traumuoto kelio **skausmo**?

❑1 Labai intensyvius veiksmus, kaip šuolius ar greitą krypties keitimą, pvz. žaidžiant krepšinį ar futbolą
❑2 Intensyvius veiksmus, pvz. kilnoti sunkumus, slidinėti ar žaisti tenisą
❑3 Vidutiniškai intensyvius veiksmus, pvz. greitai eiti ar bėgioti
❑4 Nedidelio intensyvumo veiksmus, pvz. eiti normalia sparta
❑5 Negaliu atlikti nei vieno iš anksčiau nurodytų veiksmų, kadangi dabar man labai skauda kelį

2. Per pastarąsias 4 savaites arba nuo patirtos kelio sąnario traumos, kiek laiko skaudėjo traumuotas kelio sąnarys?

	0	1	2	3	4	5	6	7	8	9	10	
**Niekada neskaudėjo**	❑	❑	❑	❑	❑	❑	❑	❑	❑	❑	❑	**Skaudėjo visą laiką**

3. Kaip stipriai šiandien skauda traumuotas kelio sąnarys?

	0	1	2	3	4	5	6	7	8	9	10	
**Visai neskauda**	❑	❑	❑	❑	❑	❑	❑	❑	❑	❑	❑	**Skauda taip, kad sunku pakelti skausmą**

4. Per pastarąsias 4 savaites arba nuo patirtos traumos, kaip sunku **buvo judinti arba lankstyti** traumuotą kelio sąnarį?

❑1 Visai nesunku
❑2 Šiek tiek sunku
❑3 Vidutiniškai sunku
❑4 Labai sunku
❑5 Ypač sunku

5. Per pastarąsias 4 savaites arba nuo patirtos traumos, kiek **sutinęs** buvo traumuotas kelio sąnarys?

- ❑1 Visai nesutinęs
- ❑2 Šiek tiek sutinęs
- ❑3 Vidutiniškai sutinęs
- ❑4 Labai sutinęs
- ❑5 Ypač sutinęs

6. Jei tavęs paprašytų atlikti toliau nurodytus veiksmus, nurodyk intensyviausius veiksmus, kuriuos galėtum atlikti šiandien, nesukeliant matomo traumuoto kelio sąnario **tinimo**?

- ❑1 Labai intensyvius veiksmus, kaip šuolius ar greitą krypties keitimą, pvz. žaidžiant krepšinį ar futbolą
- ❑2 Intensyvius veiksmus, pvz. kilnoti sunkumus, slidinėti ar žaisti tenisą
- ❑3 Vidutiniškai intensyvius veiksmus, pvz. greitai eiti ar bėgioti
- ❑4 Nedidelio intensyvumo veiksmus, pvz. eiti normalia sparta
- ❑5 Negaliu atlikti nei vieno iš anksčiau nurodytų veiksmų, kadangi mano kelis yra sutinęs net ilsintis

	**Taip**	**Ne**
7. Per pastarąsias 4 savaites arba nuo patirtos kelio sąnario traumos, ar traumuotas kelio sąnarys buvo **užstrigęs (užsikirtęs)** taip, kad negalėjai jo lankstyti?	❑1	❑2
8. Per pastarąsias 4 savaites arba nuo patirtos kelio sąnario traumos, ar patyrei pojūtį, kad tavo kelio sąnarys **stringa (kliūna)**, tačiau vis tiek galėjai jį lankstyti?	❑1	❑2

9. Jei tavęs paprašytų atlikti toliau nurodytus veiksmus, nurodyk intensyviausius veiksmus, kuriuos galėtum atlikti šiandien be pojūčio, **kad traumuotas kelio sąnarys neišlaikys manęs ar nefunkcionuos**?

- ❑1 Labai intensyvius veiksmus, kaip šuolius ar greitą krypties keitimą, pvz. žaidžiant krepšinį ar futbolą
- ❑2 Intensyvius veiksmus, pvz. kilnoti sunkumus, slidinėti ar žaisti tenisą
- ❑3 Vidutiniškai intensyvius veiksmus, pvz. greitai eiti ar bėgioti
- ❑4 Nedidelio intensyvumo veiksmus, pvz. eiti normalia sparta
- ❑5 Negaliu atlikti nei vieno iš anksčiau nurodytų veiksmų, kadangi jaučiu, kad traumuotas kelio sąnarys neišlaikys manęs ant kojų

SPORTINĖ VEIKLA

10. Kokius intensyviausius veiksmus gali atlikti reguliariai **(Ilgą laiką) traumuotu keliu**?

- ❑1 Labai intensyvius veiksmus, kaip šuolius ar greitą krypties keitimą, pvz. žaidžiant krepšinį ar futbolą
- ❑2 Intensyvius veiksmus, pvz. kilnoti sunkumus, slidinėti ar žaisti tenisą
- ❑3 Vidutiniškai intensyvius veiksmus, pvz. greitai eiti ar bėgioti
- ❑4 Nedidelio intensyvumo veiksmus, pvz. eiti normalia sparta
- ❑5Dažniausiai negaliu atlikti nei vieno iš anksčiau nurodytų veiksmų

11. Ar traumuotas kelio sąnarys įtakoja tavo gebėjimą:

		**Ne, visai ne**	**Taip, šiek tiek**	**Taip, vidutiniškai**	**Taip, labai**	**Negaliu**
a.	Lipti laiptais į viršų?	1	2	3	4	5
b.	Lipti laiptais žemyn?	1	2	3	4	5
c.	Atsiklaupti ant traumuoto kelio?	1	2	3	4	5
d.	Pritūpti kaip beisbolo gaudytojas?	1	2	3	4	5
e.	Sėdėti kėdėje sulenkus kelius ir remiantis visa pėda į grindis?	1	2	3	4	5
f.	Pasikelti nuo kėdės?	1	2	3	4	5
g.	Bėgti?	1	2	3	4	5
h.	Pašokti ir nusileisti ant traumuoto kelio?	1	2	3	4	5
i.	Staigiai pradėti judėti ir sustoti?	1	2	3	4	5

FUNKCIJA

12. Kaip gerai funkcionavo tavo kelio sąnarys **iki patiriant traumą**?

**Negalėjau nieko daryti**	0	1	2	3	4	5	6	7	8	9	10	**Galėjau daryti viską, ką norėjau**
	❑	❑	❑	❑	❑	❑	❑	❑	❑	❑	❑	

13. Kaip gerai funkcionuoja kelio sąnarys **šiuo metu**?

**Aš negaliu nieko daryti**	0	1	2	3	4	5	6	7	8	9	10	**Aš galiu daryti viską, ką noriu**
	❑	❑	❑	❑	❑	❑	❑	❑	❑	❑	❑	

14. Kas užpildė klausimyną? ❑1 Vaikas savarankiškai ❑2 Vaikas su tėvų / suaugusiųjų pagalba

15. Klausimyno užpildymo data __ __ / __ __ / __ __ __ __
MMMM MM DD
